# Monitoring the Transcriptional Activity of Human Endogenous Retroviral HERV-W Family Using PNA Strand Invasion into Double-Stranded DNA

**DOI:** 10.1007/s12033-017-0057-0

**Published:** 2018-01-08

**Authors:** Grzegorz Machnik, Estera Skudrzyk, Łukasz Bułdak, Jarosław Ruczyński, Agnieszka Kozłowska, Piotr Mucha, Piotr Rekowski, Witold Szkróbka, Marcin Basiak, Aleksandra Bołdys, Helena Sławska, Bogusław Okopień

**Affiliations:** 1 Department of Internal Medicine and Clinical Pharmacology, School of Medicine in Katowice, Medical University of Silesia, Medyków 18, 40-752 Katowice, Poland; 2 Faculty of Chemistry, University of Gdańsk, Wita Stwosza 63, 80-308 Gdańsk, Poland; 3 Department of Gynaecology, Obstetrics and Oncological Gynaecology, Medical University of Silesia, Batorego 15, 41-902 Bytom, Poland

**Keywords:** Peptide nucleic acid (PNA), Human endogenous retroviruses, Multiple sclerosis-associated retrovirus (MSRV), Quantitative methods, Polymerase chain reaction (PCR), Strand invasion

## Abstract

In the presented assay, we elaborated a method for distinguishing sequences that are genetically closely related to each other. This is particularly important in a situation where a fine balance of the allele abundance is a point of research interest. We developed a peptide nucleic acid (PNA) strand invasion technique for the differentiation between multiple sclerosis-associated retrovirus (MSRV) and ERVWE1 sequences, both molecularly similar, belonging to the human endogenous retrovirus HERV-W family. We have found that this method may support the PCR technique in screening for minor alleles which, in certain conditions, may be undetected by the standard PCR technique. We performed the analysis of different ERVWE1 and MSRV template mixtures ranging from 0 to 100% of ERVWE1 in the studied samples, finding the linear correlation between template composition and signal intensity of final reaction products. Using the PNA strand invasion assay, we were able to estimate the relative ERVWE1 expression level in human specimens such as U-87 MG, normal human astrocytes cell lines and placental tissue. The results remained in concordance with those obtained by semi-quantitative or quantitative PCR.

## Introduction

Detection or semi-quantitative estimation of specific sequence type (e.g., a mutated allele) in biological specimens is commonly required in genomic approaches. Until now, several techniques that meet these criteria have been well described. These methods, basically employing the principles of polymerase chain reaction (PCR), represent diverse modifications of the classical process described for the first time by Mullis et al. [1]. Modern techniques, both qualitative and quantitative, frequently utilize fluorescent signal derived either from labeled oligonucleotide(s) or from dsDNA intercalators such as SYBR Green I. These so-called *real-time PCR* (rtPCR) methods can be used for virtually all purposes wherever a certain sequence is to be detected or quantified [2, 3]. However, some authors discussed the limitations of rtPCR techniques. They argued that their main disadvantage is the inability to detect the mutated variant if a wild-type allele occurs in a significant excess over the sequence of interest [4, 5]. Even the most commonly used Sanger’s sequencing method, which has been perceived as a gold standard in the mutation detection approach, displays relatively low sensitivity of detection of a minor target in mixed samples [6]. This fact implicates the possible failure of determination of rare mutants during sample processing [7]. Particularly important hazard of false-negative results obtainment can take place in tumor samples, e.g., in screening for epidermal growth factor receptor (EGFR) mutations in non-small cell lung cancer (NSCLC) patients or in clinical virology [8, 9]. It has been estimated that the DNA of interest must comprise at least 25% of the total DNA to be easily detected by means of Sanger sequencing [6, 10]. To omit the risk of false-negative results, the addition of oligonucleotides that clamp the amplification of wild-type DNA sequences may be utilized. This can increase the sensitivity to the level, when only 1% of mutated DNA is present in a total DNA in the sample [11]. Some synthetic oligonucleotides, such as locked nucleic acid (LNA) or peptide nucleic acid (PNA) with improved properties compared to the naturally occurring DNA, displayed high discriminatory potential [12]. PNA molecule is also prone to modifications that can further improve its DNA-binding abilities [13].

Peptide nucleic acid (PNA), a fully synthetic oligonucleotide analogue, exhibits higher affinity for complementary DNA, RNA or PNA sequences than natural nucleic acids [14, 15]. On the other hand, if a base-pair mismatch occurs in the template DNA at any position, the binding strength of such DNA/PNA complex decreases dramatically. This feature makes PNA very suitable for single-nucleotide polymorphism (SNP) analysis [16].

Another unique feature of PNA, which clearly distinguishes it from any other synthetic DNA-binding molecules, is its ability to invade the target sites in double-stranded DNA (dsDNA). This phenomenon, termed *strand invasion*, if directed only by sequence-specific PNA, is intrinsically poorly efficient and occurs only under certain conditions, e.g., in the presence of purine- and pyrimidine-rich targets [17]. In order to gain flexibility in clinical conditions, more universal procedure must be employed to overcome above-mentioned limitation. Takumi Ishizuka and co-workers, in a number of publications, proposed the addition of a single-stranded DNA-binding protein (SSB) to the PNA-directed strand invasion assay to improve the reaction efficiency [18–20]. Due to PNA-to-DNA-binding characteristics, a strand invasion phenomenon would allow an analysis of genetic polymorphisms in a variety of assays and studies [16, 21, 22]. The possible recognition mechanisms and some applications of peptide nucleic acids that target double-stranded DNA have been comprehensively elaborated by Li et al. [23].

In PNA-directed, SSB-assisted strand invasion assay, the efficiency of strand displacement relies on several factors including: (1) PNA-to-DNA ratio; (2) SSB amount that is used to facilitate the double-strand invasion; (3) The length of PNA oligomer; (4) The complementarity between PNA and DNA template to be invaded [19]. The visualization of a PNA strand invasion phenomenon was achieved by polyacrylamide gel mobility shift assay, as described previously [21, 24]. The principle of this assay is based on the observation that double-stranded DNA, being relaxed in the presence of complementary PNA, migrates slower than its double stranded, non-altered counterpart. Moreover, if the process is effective enough, all initially double-stranded DNA converts into relaxed, single-stranded form. That process is well visible on the polyacrylamide gel by the appearance of a new band (which indicates the presence of single-stranded DNA), while double-stranded DNA becomes no more visible. After staining, the intensities of both ss- and dsDNA bands reflect the effectiveness of a strand invasion. In our work, we have elaborated on the relevancy between the strand invasion efficiency and initial template DNA amount in the investigated sample. Moreover, as PNA oligomers offer the capability for the detection of an allele-specific DNA template, we used the PNA-directed strand invasion assay for the determination of certain sequences in the biological samples. For the experimental purposes, we have chosen specific sequences belonging to human endogenous retroviruses (HERVs) as endogenous retroviruses have been of special interests for our research group for several years [25–28].

## Materials and Methods

### Sequence Selection for Analysis

Sequences belonging to the human endogenous retroviral HERV-W family were obtained from GenBank database and analyzed using EMBOSS software package [29] These sequences encode for envelope (*env)* proteins that have been implicated to play the most significant role in a number of medical conditions. Four sequences from different loci were taken into consideration. The comparison of data was carried out using Emma software, a Multiple Sequence Alignment ClustalW wrapper from EMBOSS package. The most appropriate target site for the hybridization of PNA probe was designed within a highly conserved region.

Polymerase chain reaction (PCR) was performed using oligonucleotide primer pair (HERV_F and HERV_R) that flanks the PNA target regions giving a PCR product of 270 base pair (ERVWE1_270_), Table 1. The length of this dsDNA molecule corresponds to the fragment that has been previously reported to be optimal in PNA-mediated strand invasion approaches [19].

**Table 1:** Oligonucleotides used in this work

Oligonucleotide/PNA name	Oligonucleotide/PNA sequence	Product length [base pairs]	Remarks
HERV_F HERV_R	5′-TGCCCCATCGTATAGGAGTC-3′ 5′-CGGGTGAGTTGGGAGATTAC-3′	270	HERV-W-specific, for cloning and for PNA-mediated strand invasion
HERV_REV/0	5′-GAATTCCACCCCCATCAGACA-3′	218	For HERV-W-specific QPCR analysis
PNA(14)	N′-TACCAGTTTGGGTG-C′	–	ERVWE1-specific PNA probe for QPCR analysis
PNA(14_1)	N′- TACCAGTTTGGGTGA-C′	–	ERVWE1-specific PNA probe for strand invasion assay
ACTB_F ACTB_R	5′-TCATGAAGTGTGACGTGGACATC-3′ 5′-CAGGAGGAGCAATGATCTTGATCT-3′	156	The reference gene for PCR/QPCR analyses

### Polymerase Chain Reactions, Mutagenesis and Cloning

For cloning procedures, human genomic DNA was extracted from placental tissue specimens obtained from the Department of Gynaecology, Obstetrics and Oncological Gynaecology, Medical University of Silesia, Bytom, Poland. The ERVWE1 DNA fragment amplification, mutagenesis, and subsequent cloning of DNA fragments into pSC-B-amp/kan plasmid vector (Agilent Technologies Inc. Santa Clara, CA, USA) were optimized by our research group and described previously [30]. The resulting sequence, ERVWE1_270_, represent a gene fragment encoding syncytin-1, a glycoprotein that plays an important role in placental morphogenesis [31]. In the next step, three point mutations were introduced into the selected positions in the ERVWE1_270_ PCR product. To do this, a site-directed mutagenesis procedure was performed according to the “megaprimer” technique developed by Brøns-Poulsen and co-workers [32]. Detailed protocol has been published in our previous paper [33]. After mutagenesis, ERVWE1 gene fragment became identical with that of multiple sclerosis-associated retrovirus (MRSV) and was consequently denoted as MSRV_270_. Finally, ERVWE1_270_ and MSRV_270_ amplimers were cloned into plasmid vector pSC-B-amp/kan using StrataClone Ultra Blunt PCR Cloning Kit according to the manufacturer’s instruction (Agilent Technologies Inc. Santa Clara, CA, USA). The identity of cloned fragments were confirmed through DNA sequencing based on Sanger’s dideoxynucleotide method with the use of insert-specific primers, i.e., HERV_F/HERV_R (sequencing was performed by Genomed SA, Warsaw, Poland). The comparative analysis showed 100% identity with the reference sequences (GenBank: AF208161 or AF331500, for ERVWE1 and MSRV, respectively). Plasmid DNA extracts were quantified using SYBR Green 1 fluorescent dye (Life Technologies, Warsaw, Poland) with the λ phage DNA as a standard reference (Thermo Fermentas, Vilnius, Lithuania). Based on the plasmid DNA concentration, the molecular mass was calculated using dsDNA copy number calculator (http://cels.uri.edu/gsc/cndna.html).

### Synthesis of Peptide Nucleic Acid (PNA) Oligomers

Peptide nucleic acid oligomers were synthesized and purified at the Faculty of Chemistry, University of Gdańsk, Poland. PNA monomers for synthesis were purchased from Panagene (Daejeon, Korea). PNA oligomers were synthesized using a Labortec AG SP-650 Peptide Synthesizer on Fmoc-XAL-PEG-PS resin, capacity of 0.18 mmol/g (Merck KGaA, Darmstadt, Germany). All Fmoc/Bhoc protected PNA monomers were assembled as active derivatives with the use of a threefold molar excess. Monomers were activated with the use of a 2-(1H-7-azabenzotriazole-1-yl)-1,1,3,3-tetramethyluronium hexafluorophosphate (HATU)/4-methylmorpholine (NMM)/2,6-lutidine (0.7:1:1.5) mixture in *N*,*N*-dimethylformamide (DMF)/*N*-methyl-2-pyrrolidone (NMP) (1:1, *v/v*) solution mixture for 30 min. Deprotection of the Fmoc (9-fluorenylmethoxycarbonyl) group was conducted with 20% piperidine in DMF for 2 cycles (2 × 2 min). Cleavage and deprotection of the Bhoc (benzhydryloxycarbonyl) group of the immobilized PNA were performed by treatment with a trifluoroacetic acid (TFA)/m-cresol 95:5 (*v/v*) mixture for 30 min. The obtained crude PNA oligomers were lyophilized, purified and analyzed using the reverse-phase, high-performance liquid chromatography (RP-HPLC) as well as MALDI-TOF (matrix-assisted laser desorption/ionization-time of flight) mass spectrometry. All analytical RP-HPLC separations were performed on a Phenomenex Kinetex XB-C18 (4.6 × 150 mm, 5 µm particle size) using an Agilent 1100 System and several gradient methods. The mobile phase consisted of 0.08% TFA in acetonitrile (solvent A) and 0.1% TFA in water (solvent B). The column was maintained at ambient temperature. The flow rate was 1 ml/min, and the eluted solution was monitored with a UV detector at 254 nm. Semi-preparative RP-HPLC purifications of synthesized products were performed at ambient temperature on a Kromasil C8 column (16 × 250 mm, 5 µm particle size) using a Knauer system and several gradient methods. The mobile phase was the same as for analytical RP-HPLC, but the flow rate was 4.5 ml/min. Fractions which exhibit the purity greater than 98% were collected and lyophilized. Purified PNA was characterized with MALDI-TOF mass spectrometry (Bruker, BIFLEX III). Molecular masses of the obtained PNA oligomers are presented in Table 2.

**Table 2:** Characteristics of synthesized PNA oligomers

Name	PNA sequence	Molecular mass (Da)		Purity (%)
		Calculated	Found	
PNA14	N′-TACCAGTTTGGGTG-C′	3857.6	3857.3 [M + H]^+^	99.2
PNA14(1)	N′-TACCAGTTTGGGTGA-C′	4133.9	4135.1 [M + H]^+^ 2068.9 [M + 2H]^2+^	98.6

### PNA-Mediated, Double-Strand DNA Invasion Assay

Strand invasion conditions were optimized experimentally; details have been described in our previous report [34]. The optimal reaction conditions were as follow: ERVWE1_270_ or MSRV_270_ template DNA: 34 nM; PNA(14_1): 100 nM; SSB: 2.2 ng of total protein per reaction, TE Buffer (Tris–HCl: 10 mM, EDTA: 1 mM, pH = 6.8): up to 10 μl. Total reaction volume: 10 μl. Reaction mixtures were incubated in a dry heating block at 37 °C for 90 min and then centrifuged briefly and cooled on ice. Single-Stranded DNA-Binding Protein, SSB (Cat. No. M3011) was purchased from Promega GmbH (Mannheim, Germany).

The linear character of PNA-mediated strand invasion in the increasing template concentrations was examined similarly to the method described by Jeong et al. [35]. Briefly, plasmid (pSC-B plasmid, Agilent Technologies, Warsaw, Poland) that contained cloned ERVWE1 PCR product (ERVWE1_270_) was serially diluted with the plasmid containing MSRV sequence fragment (MSRV_270_). The proportions of ERVWE1_270_ were adjusted to 100, 80, 60, 40, 20, 5, 1 and 0% (ERVWE1_270_/MSRV_270_ ratio). The same preparations were used in the PNA-mediated strand invasion assay as well as in further polymerase chain reaction (PCR) analyses.

### DNA Electrophoresis and Blotting

The reaction products of PNA strand invasion were loaded onto 6%, 1-mm-thick polyacrylamide gel (29:1 acrylamide/Bis-acrylamide ratio) and resolved for 2.5 h at 100 V. After electrophoresis, gels were stained for 10 min in the solution containing 1× concentrated SYBR Green I nucleic acid gel stain (Sigma-Aldrich Co, Poznań, Poland), then washed with high-pure water for 5 min, and digitalized using Gel Logic 100 Imaging System (Eastman Kodak Company, Rochester, NY, USA). Integrated optical density (IOD) values for every DNA band were calculated using ImageJ, v. 1.41 software [36].

After staining and digitalizing, DNA was electrotransferred onto a positively charged, nylon blotting membrane (Hybond-N+, GE Healthcare Life Sciences, Warsaw, Poland). Electroblotting was carried out overnight in 1× TBE buffer at constant current of 100 mA in a TE22 Electroblotter (Hoefer Inc, Holliston, MA, USA). DNA was fixed on the membrane by baking it at 80 °C for 1 h in a sterilization oven. An ERVWE1-specific, biotinylated RNA probe was synthetized using T3 RNA polymerase and Biotin RNA Labeling Mix (Roche Diagnostics, Warsaw, Poland); XbaI-linearized pSC-B-ERVWE1_270_ plasmid served as a template in transcription reaction. Hybridization was performed in a roller bottle overnight at 55 °C using 2.5 μl of RNA probe diluted in 4 ml of hybridization buffer (Minidizer Hybridizer, UVP, LLC, Upland, CA, USA). All subsequent steps in hybridization procedure were performed according to the manufacturers protocols (North2South Chemiluminescent Hybridization and Detection Kit, Pierce Biotechnology, Rockford, IL, USA). Chemiluminescent signal from ERVWE1-specific probe was detected by CCD camera in ChemiDoc-It Imager darkroom (UVP, LLC, Upland, CA, USA). Integrated optical density (IOD) values of bands were calculated in the same way as for DNA in the gels.

### Polymerase Chain Reaction (PCR) and Quantitative Polymerase Chain Reaction (QPCR)

PCR reaction mixture contained 12.5 μl of 2× TaKaRa Premix Taq DNA Polymerase (Takara Bio Europe, Saint-Germain-en-Laye, France), 200 nM of each HERV_F and HERV_REV/0 primers and 4 μM of PNA probe (PNA_14) or an equivalent of 5% dimethylsulfoxide/water in the control reactions. Plasmid dilutions containing different ratios of ERVWE1/MSRV templates were analyzed in duplicates using 2 μl of template solutions. An additional PNA annealing step was introduced into the typical, three-step PCR because hybridization temperatures of PNA and primers differed significantly. An optimal thermal conditions were set as follows: 95 °C/5 min, 26 repeats of (95 °C/30 s, 63 °C/50 s, 58 °C/30 s, 72 °C/40 s) and 72 °C/10 min. Reactions were set for only 26 cycles in order to achieve the logarithmic increment of the amplimer number. Reaction products were then electrophoresed and used for further semi-quantitative measurements.

Detection and quantitation of human endogenous retrovirus W-family (HERV-W) sequences in RNA (cDNA) from normal human astrocytes (NHA), U-87 MG astrocytoma cells, and several samples of human placental tissue were accomplished by means of quantitative polymerase chain reaction (QPCR). HERV-W sequences were obtained using universal primers for ERVWE1 and MSRV. The complete description of that sequence-specific assay can be found in our previous paper [33]. All analyses were performed on Roche LightCycler 480 Real-Time PCR system (Roche Diagnostics, Basel, Switzerland).

Total RNA was extracted from NHA-normal human astrocytes (Lonza Ltd, Warsaw, Poland), U-87 MG astrocytoma cell line (Sigma-Aldrich Co, Poznań, Poland), and human placental tissue (four independent samples). One milliliter of TRI reagent (MRC Inc., Cincinnati, OH, USA) was used to lyse cells scrapped from tissue culture plates of 35 mm diameter or 50 mg of placental tissue. The latter sample type was additionally homogenized by means of a rotor/stator, hand-held homogenizer (IKA-Werke GmbH & Co. KG, Staufen, Germany). Subsequent RNA extraction steps were performed according to the manufacturer’s protocol. Finally, total cellular RNA was dissolved in 150 μl of nuclease-free water. After extraction, 2 μg of total RNA was digested with DNase-I (RNase-free, Life Technologies, Warsaw, Poland) in order to remove any possible contamination with residual DNA. Then, 1 μg of DNA-free RNA was reverse-transcribed in the final volume of 20 μl. Reaction mix was subsequently diluted five times with water as stated in the manufacturer’s protocol (GoScript Reverse Transcription System, Promega GmbH, Germany).

Reaction mixtures contained 10 μl of GoTaq^®^ qPCR Master Mix (Promega GmbH, Mannheim, Germany), 0.2 μM of each primer (HERV_F and HERV_REV/0, respectively), 4 μM of PNA probe (PNA_14). Three microliters of the diluted reverse transcription reaction mixture was used as template. Every sample was analyzed in triplicates, parallel with control reaction without PNA probe. QPCR thermal profile was set as follows: 95 °C/5 min, then 35 cycles of 95 °C/30 s, 63 °C/50 s, 58 °C/30 s, 72 °C/40 s with signal acquisition for SYBR Green I dye at the end of each primer annealing step (please note the unusual, four-step thermal profile with an additional annealing step that is necessary for the annealing of PNA probe). The specificity of QPCR was confirmed after reaction by melting-curve analysis as well as by agarose gel electrophoresis.

## Results

As we previously have found and published, PNA(14) molecules efficiently act as strand invaders against the double-stranded, HERV-W *env*-derived PCR products at concentrations > 200 nM [33]. Under specific conditions the selective inhibition against ERVWE1_270_ but not MSRV_270_ was achieved. The proper reaction parameters, such as PNA-to-template ratio, SSB concentration, and buffer composition, strongly influenced the strand invasion of dsDNA by PNA. In our setting the highest strand invasion efficiency was achieved under the conditions specified in Materials and Methods section (DNA concentration: 34 nM; PNA(14_1) concentration: 100 nM; SSB concentration: 2.2 ng of total protein per reaction, reaction volume of 10 μl in TE buffer, pH = 7.5). Details were provided in our previous paper [33].

**Fig. 1:**
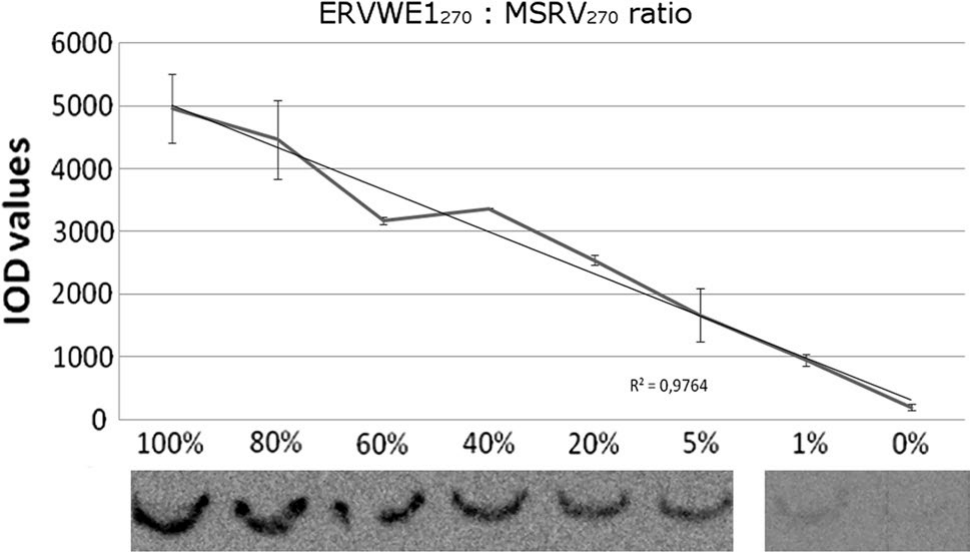
The formation of single-stranded DNA caused by the PNA strand invasion maintains the linear intensity within a range of initial amount of ERVWE1 DNA in respect to bulk MSRV DNA. This linearity was observed in the range of 100, 80, 60, 40, 20, 5, 1 and 0% (ERVWE1/MSRV ratio). The potency of PNA strand invasion was measured as strength of chemiluminescent signal from ssDNA-specific, biotin-labeled RNA probe. The analysis was performed in triplicate, and an average value for each point was calculated

We observed that the formation of single-stranded DNA as a result of PNA strand invasion maintained the linearity over a wide range of template composition (ERVWE1_270_/MSRV_270_ ratios: 100, 80, 60, 40, 20, 5, 1 and 0%) (Fig. 1). The efficiency of strand invasion was measured as a chemiluminescent signal from specific RNA probe that recognized and binded to single-stranded DNA.

**Fig. 2:**
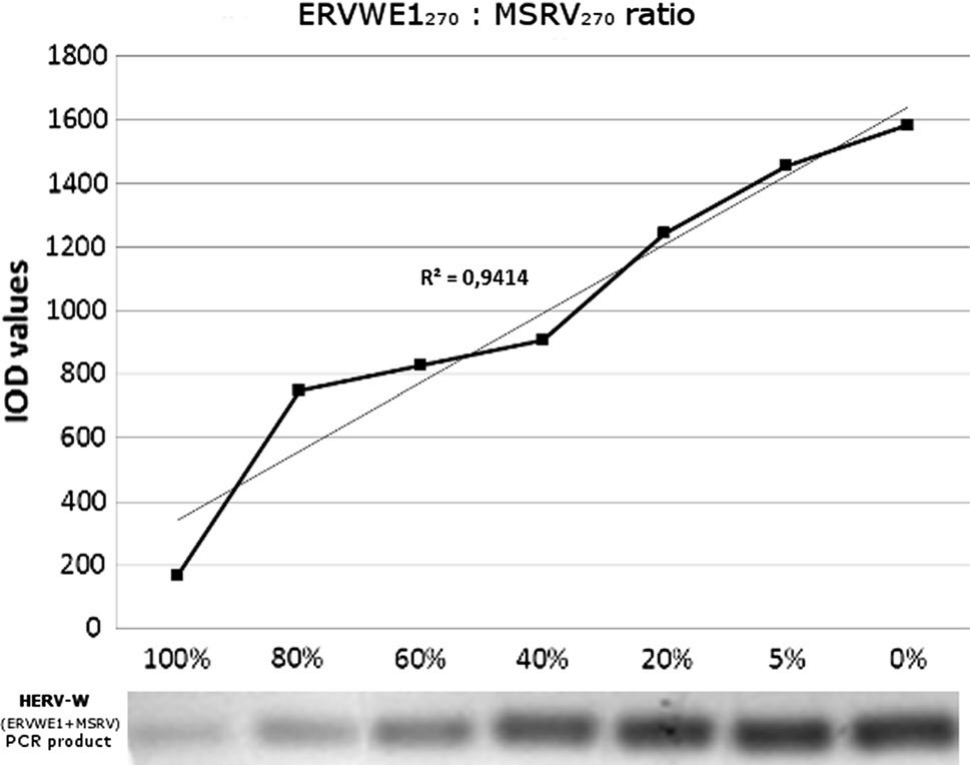
Semi-quantitative polymerase chain reaction (PCR) specific for HERV-W (ERVWE1 + MSRV) template. Similarly to the PNA strand invasion, the linearity of this assay was observed for the concentrations of 100, 80, 60, 40, 20, 5 and 0%. The intensity of reaction was assessed by a fluorescent signal from ethidium-bromide labeled PCR products resolved on an agarose gel. In order to achieve the logarithmic increment of the amplimers’ number during reaction, PCR was set for 26 thermal cycles only. A correlation coefficient between PNA strand invasion (Fig. 1) and PCR (Fig. 2) reached a value of *R* = 0.943 indicating high correlation between compared methods

Parallelly, in PCR that served as a reference method we also noted a linear increment in bands optical density (IOD) values of HERV-W family (ERVWE1 + MSRV) together with decreased concentration of ERVWE1 DNA due to its exclusive inhibition by PNA, i.e., the less ERVWE1 sequences were present in the sample (concomitantly with increasing MSRV DNA concentration), the stronger band intensity was observed. This quantitative relation was maintained within a range of ERVWE1/MSRV DNA mixtures (Fig. 2). A correlation coefficient for PNA strand invasion and PCR was calculated reaching a value of *R* = 0.943 that indicates high correlation between compared methods, correlation coefficient strength according to Guilford, [37].

**Fig. 3:**
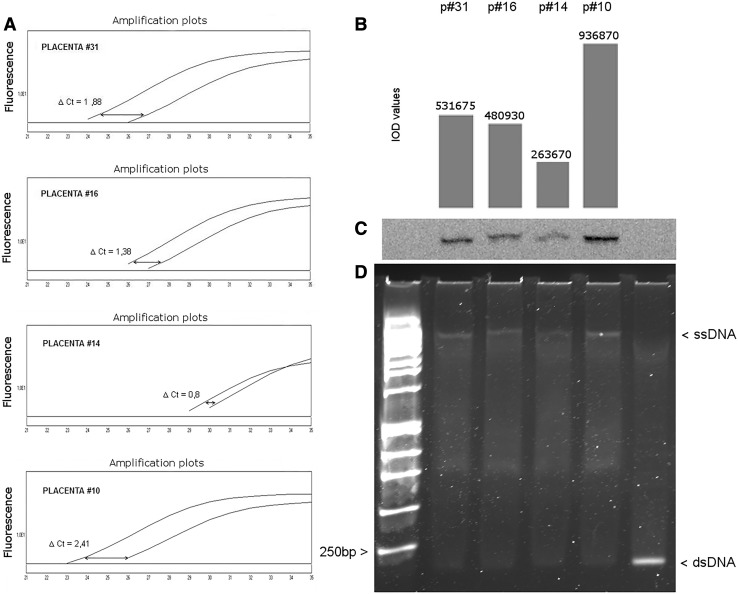
The estimation of the ERVWE1 expression level in samples obtained from different human placental tissue. **a** Real-time quantitative PCR. Reaction mixtures contained an allele-specific, peptide nucleic acid probe [PNA(14)] that inhibited amplification of ERVWE1 template while control reactions did not contain PNA(14). Higher ΔCt value represents greater amount of ERVWE1 in the sample. **b**, **c** The formation of single-stranded DNA (ssDNA) as a result of PNA strand invasion. Signal strength from ssDNA-specific, biotin-labeled RNA probe varied between particular samples indicating different amount of ERVWE1 RNA (cDNA) in the samples. **b** Integrated optical density (IOD) values calculated for the ssDNA bands presented in **c**. **d** Gel mobility shift assay visualized PNA strand invasion process in placental samples. The SYBR-I dye signal strength was measured directly in the polyacrylamide gel after electrophoresis. During strand invasion double-stranded DNA became relaxed and single-stranded (ssDNA) are formed that migrated much slower than dsDNA. The bands’ intensity reflects different reaction efficiency in assayed samples

In the next step, four samples taken from different placental tissues were analyzed in order to estimate the expression level of ERVWE1 sequences. Using PNA strand invasion and rtQPCR, we determined the levels of ERVWE1 expression in particular placental specimens. For each sample, we calculated the mean ΔCt value and the intensity of chemiluminescent signal from single-stranded DNA after PNA strand invasion. We have proven that results of the ERVWE1 expression level were similar and did not depend on the technique used for analysis (Fig. 3).

The principle of ERVWE1 quantitation by QPCR relies on the threshold cycle (Ct) parameter that is determined during reaction course. By definition, Ct is the cycle number at which the fluorescence signal crosses the background (threshold) level. The Ct value has been broadly acknowledged by researchers in indirect quantitation of initial copy number in a sample in respect to controls. In our assay, the ΔCt value for a sample is defined as follows:

$$\Delta {\text{Ct}} = {\text{Ct}}_{{(w / {\text{PNA}}\,{\text{probe}})}} - {\text{Ct}}_{{(w /o\;{\text{PNA}}\,{\text{probe}})}} ,$$

where PNA probe inhibits products amplification from ERVWE1 but not MSRV templates in the reaction. Finally, ΔCt makes a parameter that reflects the relative expression level of ERVWE1 in a sample in respect to that expression in the control into which no PNA probe has been added. As PNA probe selectively blocks the amplification from ERVWE1 template, the higher ΔCt value indicates that many ERVWE1 molecules were present in a sample compared to other HERV-W *env* sequences, e.g., MSRV. Some additional factors that could influence the QPCR efficiency (such as fluctuations in initial cDNA amount, reaction inhibitors etc.) were corrected through the simultaneous analysis of a house-keeping gene of beta-actin (ACTB) in every sample.

**Fig. 4:**
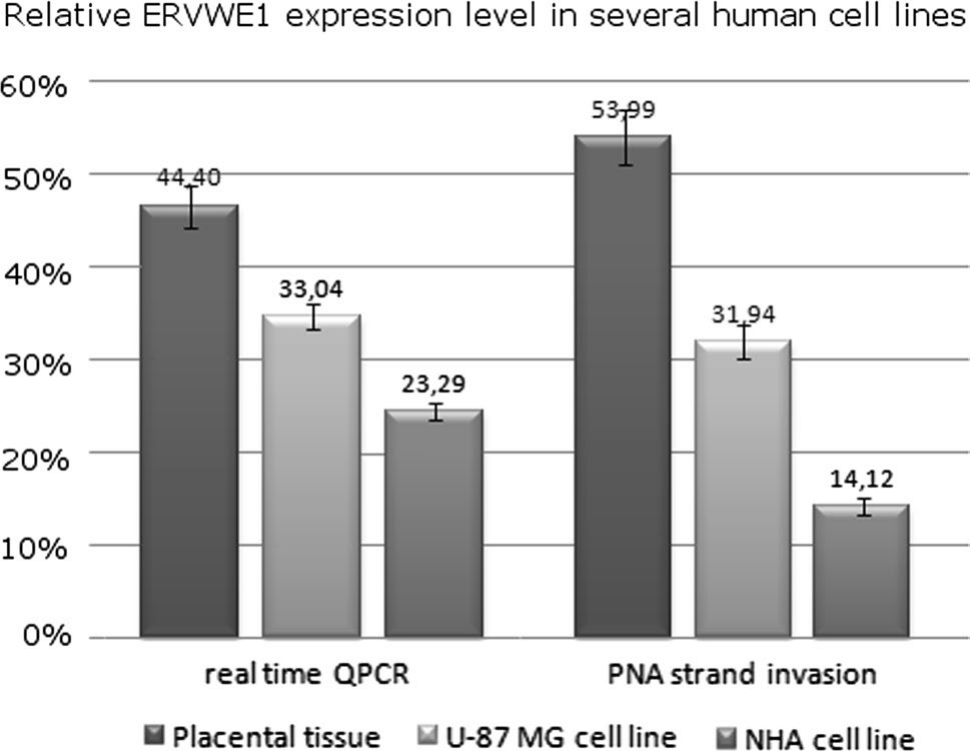
The measurement of ERVWE1 mRNA level in total RNA extracts derived from biological specimens. Normal human astrocytes (NHA), U-87 MG astrocytoma cell line and placental tissue were analyzed by means of PNA strand invasion and real-time, quantitative PCR (QPCR). The diagrams indicate that both strand invasion as well as QPCR returned similar result although they relied on different theoretic principles. It has been shown that ERVWE1 is differentially expressed in human cell-derived transcripts, where its expression was most abundant in the placental tissue

Another analysis concerned the estimation of the proportion of ERVWE1 transcripts in overall HERV-W *env* sequences present in RNA extracts from biological specimens (human cell lines and placental tissue). The estimated ratios between ERVWE1 levels in respect to the total level of HERV-W *env* RNA (cDNA) were comparable for both techniques (paired Student’s *t* test: SE = 2.74, *t* = 0.081, *p* > 0.4). This observation was true for all cell types, i.e., for normal human astrocytes (NHA), U-87 MG astrocytoma cell line, and for placental tissue (Fig. 4). As we expected, the highest expression level of ERVWE1 was observed in placental tissue. Placenta has been previously recognized as a site where ERVWE1 expression product (i.e., syncytin-1 glycoprotein) plays a significant, physiological role [38]. Real-time QPCR, as well as PNA strand invasion assay, showed that ERVWE1 comprised a significant proportion of total HERV-W *env* RNA in placenta (44 and 54%, respectively). In malignant human astrocytes (U-87 MG cell line) the ERVWE1/total HERV-W *env* expression ratio was relatively higher than in normal human astrocytes (NHA) reaching 32% (by QPCR), and 34% (by PNA strand invasion assay). For normal human astrocytes (NHA) the ERVWE1 expression values were 23 and 14%, respectively (Fig. 4). Calculations were performed using the STATISTICA v. 9.0 software (StatSoft Polska). These findings are in concordance with previously published papers describing that mRNA copy number expressed from certain HERV-W *env* genomic loci enormously increases in astrocyte cells under some pathological neurological conditions [39]. Conversely, astrocytes being in quiescent state do not display any changes in HERV-W *env* expression pattern [40].

## Discussion

Through their unique features, such as strong specificity to a chosen DNA template, peptide nucleic acids (PNAs) may pose a useful tool in the manipulation and analysis of DNA [41]. For instance, the precise DNA scission in a pre-defined site is possible through an artificial restriction DNA cutter (ARCUT) combining the PNA and a cleaving complex containing Ce(IV)/EDTA [42]. It has been also reported that PNA can facilitate the enzymatic cleavage capability of S1, mung bean, and BAL31 nucleases [43, 44]. Additionally, some enzymes, other than nucleases, can improve the versatility of PNA as well. For instance, single-stranded DNA-binding protein (SSB) promotes the invasion of PNA into double-stranded DNA at an arbitrary chosen position and that approach offers a number of potential advantages [34]. The PNA molecule, being a part of strand invasion complex, can precisely distinguish between “wild-type” and “mutated-type” sequence thereby allowing the strand unwinding exclusively within an appropriate sequence type. In this paper, we have evidenced the PNA-based assay has a quantitative potential. Currently, a polymerase chain reaction (PCR), as well as its subsequent modifications such as real-time quantitative PCR (QPCR), provides a top-cited method in genomic and gene expression studies. Therefore, we have aimed to compare the possible linearity of PNA strand invasion phenomenon with that of PCR/QPCR within a range of template concentrations. As our group has been worked with human endogenous retroviral-W family for a long time, we have decided to use those sequences as a model molecule. Since individual HERV loci (with a multiple sclerosis-associated retrovirus, MSRV, as the main example) have been suspected to display pathogenic properties, the PNA strand invasion assay may be useful in detection of possible fluctuations in that retroviral expression pattern. In search for a new and robust method to pursue our objective, we have also emphasized the potential of PNA strand invasion phenomenon for the recognition of other DNA sequences. Using PCR-based, allele-specific assays, the rare allele can often be underestimated or even missed during analysis [35, 45]. In this respect we consider the PNA-directed/SSB-assisted stand invasion assay to be a good research tool if a balance between two similar sequences in a sample has to be estimated.

Our results show that PNA-mediated/SSB-assisted unwinding of a specific DNA type maintains the linearity within a range of initial ERVWE1: MSRV DNA mixtures (Fig. 1). Similar features of peptide nucleic acid were shown in PCR using ERVWE1-specific PNA molecules (PNA(14) and PNA14_1) and universal HERV-W primers. Due to successful inhibition of ERVWE1 amplification by PNA(14), only MSRV products became exposed (Fig. 2). This allowed us to estimate the amount of ERVWE1 in selected mixtures of templates.

In biological specimens, we presented the results as a relative amount of ERVWE1 molecule that was detected in an investigated cell type in respect to an overall ERVWE1 amount detected in all samples, including different cell types and replicates. This approach allows comparing the degree of relative expression estimated by PNA strand invasion and PCR. Nevertheless, it should be kept in mind that both methods rely on different theoretic principles and thus their results are not directly comparable. QPCR provides the results as relative expression of ERVWE1 in the presence/absence of ERVWE1-inhibiting PNA (a ΔCt value) while the efficiency of PNA-mediated, SSB-assisted DNA strand invasion was measured as an optical density of chemiluminescent signal from single-stranded DNA bands after hybridization of ssDNA with a labeled probe. Therefore, we expressed the comparison of both methods as an ERVWE1 ratio that was evaluated in certain cell line, i.e., in normal human astrocytes (NHA), U-87 MG astrocytoma cells, and in placental tissue compared to total ERVWE1 expression measured in all cells (Fig. 4).

It has been documented that a broad range of disorders, including psycho-neurologic symptoms, such as multiple sclerosis, schizophrenia, certain cancer types (e.g., breast cancer), or even diseases of an auto-immunologic background (psoriasis, lupus erythematosus) correlate with altered expression pattern of human endogenous retroviruses (HERVs) [46–49]. Although HERVs have been considered as a type of “junk” DNA for a long time, recently a growing number of papers demonstrate that in numerous pathological conditions a role of different HERV families has been at least suspected [50, 51]. Mainly it has been observed as a change in mRNA copy number (either increased or decreased) of specific, particular loci of HERV families which correlates with a certain pathological condition while other HERV members remain in quiescent state.

We indicate that PNA-mediated strand invasion assay may serve as a complementation of real-time QPCR technique, especially if a relation between very similar templates is a point of interest. We proved that strand invasion phenomenon, directed by sequence-specific PNA, occurs efficiently and maintains the linearity even at low ratio of desired template (until 5% of total DNA in a sample). PCR-based techniques require at least 20% of the mutated allele contents to assure the detection rate toward that minor sequence [5].
